# Some Imperfections of Medical Education

**Published:** 1895-01-26

**Authors:** 


### Some Imperfections of Medical Education.

In the interesting address delivered by Mr. Ernest Hart before the Indian Medical Congress great stress is laid on the importance and variety of the diseases which are peculiar to tropical regions, and it is forcibly, and most opportunely, pointed out that while the great mass of the diseases met within the tropics are special and peculiar, differing *in toto* from those which are common in more temperate regions, there is no provision in the London medical schools for learning anything about them. A glance at a map of the world, shows at once the enormous extent of that inter-tropical belt, which, encircling the world, contains within itself so large a proportion of the human race and, what perhaps is of more personal interest to ourselves, embraces so many of our colonial possessions. This area swarms with Englishmen, inhabiting the ports or occupying trading stations, or engaged in developing the country, and for all these London-made doctors are required. Yet the arrangements for teaching the diseases special to tropical climates are of the most meagre description. Medicine is taught clinically in London; it is in clinical work, careful watching of cases by the bedside, that London excels, and the Colleges examine on the class of work which is current in the hospitals. General medicine and general surgery hold the field. Eyes, ears, noses, and the niceties of gynæcology, everything in fact that smacks of specialism, is tabooed, and tropical diseases, being a speciality known to the comparatively few, share the same fate. Not seen clinically, not examined upon, and therefore slurred over in lectures, not likely to occur in the practice of four out of five of the students, is it to be wondered at that these maladies are neglected by those whose sole aim in studying medicine is to get a diploma? The results are disastrous. So helpless are the men turned out by the Royal Colleges, that as soon as they are accepted by the army they have to go down to Netley to learn the work in which their daily lives will afterwards be passed. But all men do not go to Netley, and those who on the strength of their London training go out to hot climates and try to do their best soon see with what difficulties they are surrounded. Landed in the midst of malignant malarial disease how willingly would they barter some of their knowledge of the minute anatomy of the spinal cord, of the localisation of function in the brain, or even of the varied virtues of the hundred and one antiseptics used in the London hospitals; how willingly would they exchange all this, and more, for some notion as to what to do. They find themselves surrounded with types of disease many of which are but casually mentioned in the text-books, some of them not mentioned at all, things they have never seen, have hardly heard of, and certainly have not been examined upon, and yet upon their treatment and upon their judgment in the administration of potent remedies hang the lives of their patients.

It must be remembered that in some of the malarial cases the disease is apt to be acute, sudden, and fatal in a few hours if relief cannot be obtained; but the information contained in the ordinary text-books concerning them is of the most meagre description. Nor can the teaching in the schools on tropical diseases be looked on as any more satisfactory. From a cosmopolitan point of view the London schools are mere provincial institutions, places in which men are given certain opportunities for picking up knowledge of the common diseases of the locality, but by no means places where men are taught the science of medicine as a whole. Even the specialties in vogue in this country are not taught by leading specialists unless they happen to be on the staff of a school-hospital, and tropical disease is even more neglected.

Yet it need not be so. There are men in London who know and who could teach all about tropical diseases; but men who have passed their lives getting this knowledge have no position in London hospitals or in London schools. To get a position in a London hospital a man must begin at the beginning and work his way up, and so it happens that all the various medical schools in London are engaged in teaching each the same little circle of knowledge, some laying more stress upon this point, others upon that, but none attempting to draw to themselves the best teaching available on every subject, or to equip their students so as to be able to practice in any part of the world. Even post graduate teaching is so arranged as to fritter away the maximum of a student's time and give him in return the minimum of knowledge. It seems hopeless to expect such a degree of co-operation among the schools as to make it possible for the ordinary student, tied down as he is by his composition fee, to get the cream of the teaching to be had in London. He must take things as they come in the school that he selects. With the cream of physiology he must drink the dregs of anatomy in the one school; with the best of anatomy he must listen to the dry droning of stale medicine in another. But in post graduate teaching one might have looked for better things. There seems no reason why, for those who have finished their course, and for the immense number of strangers who congregate in London every year, the very best of London teaching should not be available at some central spot, without the weary travelling to all quarters of the town which is entailed on them at present. But no! in consequence apparently of some extraordinary jealousy among the members of the hospitals staffs, if one is to lecture so must the rest, as if the mere fact of being on the staff gave teaching power; and so instead of useful courses by modern men we find that in most places the post graduate student is expected to attend odd jumbles of heterogeneous lectures, representative of nothing but the fads and fancies of those who give them. Never was a place where the opportunities for teaching the wider aspects of medicine were so great as they are in London, and never one where they were so wasted for lack of organisation.

